# Supplementary material for: Using citation network analysis to enhance scholarship in psychological science: A case study of the human aggression literature
Source: PLoS One. 2022 Apr 21;17(4):e0266513. doi: 10.1371/journal.pone.0266513 (PMC9022888; doi:10.1371/journal.pone.0266513)
Supplement: S1 File — This file contains the following sections. Section A: list of papers in the composite ranking, ranked from 11th to 20th position; Section B: list of the top 5 papers for the communities from the 6th to the 15th; Section C: top 10 papers based on citation ranking; Section D: principal component analysis (PCA) on network metrics; Section E: network metrics. The file contains the following figures and tables. Table 5: explanation of the variance of the principal component analysis on the network metrics. Fig 6: scree plot and PCA plot. Fig 7: representation of the top 10 papers for each network metric. (PDF) [file pone.0266513.s001.pdf]

7 Supplementary material

The data was filtered by excluding the papers with less than three citations. Thus, analysis were performed on an adjacency matrix of 1345×1345 papers.

7.1 Section A: composite ranking

Papers ranked from 11th to 20th position:
11) The Effect of Video Game Competition and Violence on Aggressive Behavior: Which Characteristic Has the Greatest Influence? [2]
12) Children, adolescents, and the media. [96]
13) The Role of Perceived Injustice in the Experience of Chronic Pain and Disability: Scale Development and Validation [98]
14) Human aggression: A social-cognitive view [5]
15) Tit-for-tat: The neural basis of reactive aggression [63]
16) Neural Mechanisms of the Testosterone–Aggression Relation: The Role of Orbitofrontal Cortex [71]
17) A longitudinal study of the association between violent video game play and aggression among adolescents.[111]
18) Acceptability of workplace bullying:a comparative study on six continents. [83]
19) Aggression's typologies[85]
20) State, not trait, neuroendocrine function predicts costly reactive aggression in men after social exclusion and inclusion [45]

7.2 Section B: Communities from the 6th to the 15th

We examined only communities with at least ten papers, for a total of 15 communities.

6) PTSD:

1. The Appetitive Aggression Scale—development of an instrument for the assessment of human's attraction to violence [107]
2. Spatial Presence and Perceived Reality as Predictors of Motion-Based Video Game Enjoyment [92]
3. Feelings of revenge, retaliation motive, and posttraumatic stress reactions in crime victims [77]
4. Brain Mechanisms Underlying Reactive Aggression in Borderline Personality Disorder—Sex Matters [56]
5. When combat prevents PTSD symptoms—results from a survey with former child soldiers in Northern Uganda [108]

7) supervisor's aggression:

1. Understanding supervisor-targeted aggression: a within-person, between-jobs design [59]
2. How management style moderates the relationship between abusive supervision and workplace deviance: An uncertainty management theory perspective [100]
3. Employees' behavioral reactions to supervisor aggression: an examination of individual and situational factors [72]
4. Emotions, Violence and Counterproductive Work Behavior [94]
5. Psychosocial predictors of supervisor-, peer-, subordinate-, and service-provider-targeted aggression [60]

8) Social pain and exclusion:

1. The interactive effect of social pain and executive functioning on aggression: an fMRI experiment [30]
2. It's the thought that counts: The role of hostile cognition in shaping aggressive responses to social exclusion [37]
3. Testing the reliability and validity of different measures of violent video game use in the United States, Singapore, and Germany [18]
4. Short fused? associations between white matter connections, sex steroids, and aggression across adolescence [80]
5. What Variables Are Associated With an Expressed Wish to Kill a Doctor in Community and Injured Patient Samples? [16]

9) Oxytocin and aggression:

1. Children, adolescents, and the media [95]
2. Effects of oxytocin on women's aggression depend on state anxiety [23]
3. When the Love Hormone Leads to Violence Oxytocin Increases Intimate Partner Violence Inclinations Among High Trait Aggressive People [38]
4. R-rated Movie Viewing, Growth in Sensation Seeking and Alcohol Initiation: Reciprocal and Moderation Effects [97]
5. Media violence and children: A complete guide for parents and professionals [47].

10) Injustice:

1. The Role of Perceived Injustice in the Experience of Chronic Pain and Disability: Scale Development and Validation [98]

2. An actor-focused model of justice rule adherence and violation: the role of managerial motives and discretion [91]

3. Excessive users of violent video games do not show emotional desensitization: an fMRI study [99]

4. Retaliation as a response to procedural unfairness: a self-regulatory approach [14]

5. An experimental investigation of the effect of a justice violation on pain experience and expression among individuals with high and low just world beliefs [101]

11) Alcohol and aggression:

1. Short-term and long-term effects of violent media on aggression in children and adults [21]

2. Workers' perception of workplace bullying: A cross-cultural study [40]

3. The disguise of sobriety: unveiled by alcohol in persons with an aggressive personality [51]

4. Alcohol and aggression without consumption. Alcohol cues, aggressive thoughts, and hostile perception bias [9]

5. Self-Regulation and Self-Regulation Failure [54]

12) No title:

1. Precarious manhood [106]

2. Gender linked Differences in the Toys, Television Shows, Computer Games, and Outdoor Activities of 5 to 13 year old Children [29]

3. Transformational Leadership and Childrens' Aggression in Team Settings: A Short-Term Longitudinal Study [102]

4. Virtual reality applications in forensic psychiatry [10]

5. An experimental investigation of the interpersonal ramifications of lateness to workplace meetings [76].

13) Anger:

1. Emotion differentiation moderates aggressive tendencies in angry people: A daily diary analysis [82]

2. Response speed as an individual difference: Its role in moderating the agreeableness–anger relationship [15]

3. Every cloud has a silver lining: interpersonal and individual differences determinants of anger-related behaviors [64]

4. Rejected by peers-attracted to antisocial media content: rejection-based anger impairs moral judgment among adolescents [81]

5. Beat them or ban them: the characteristics and social functions of anger and contempt [42].

14) Literature review guidelines:

1. Understanding frameworks and reviews: a commentary to assist us in moving our field forward by analyzing our past [90]

2. Is aggression in children with behavioural and emotional difficulties associated with television viewing and video game playing? A systematic review [73]

3. Writing Qualitative IS Literature Reviews - Guidelines for Synthesis, Interpretation, and Guidance of Research [89]

4. What literature review is not: diversity, boundaries and recommendations [104]

5. A Hermeneutic Approach for Conducting Literature Reviews and Literature Searches [13].

15) Horses and aggression:

1. Partners with Bad Temper: Reject or Cure? A Study of Chronic Pain and Aggression in Horses [44]

2. How to keep your horse safe? An epidemiological study about management practices [68]

3. Geometric morphometrics as a tool for improving the comparative study of behavioural postures [43]

4. Comparison of clinical examinations of back disorders and humans' evaluation of back pain in riding school horses [67]

5. Do horses with poor welfare show 'pessimistic' cognitive biases? [55]

7.3 Section C: Citation ranking

1) Violent video game effects on aggression, empathy, and prosocial behavior in Eastern and Western countries: A meta-analytic review [6] (comm. "media & video games")
2) The influence of media violence on youth [4] (comm. "media & video games")
3) Violent video games and hostile expectations: A test of the general aggression model [19] (comm. "media & video games")
4) The effects of prosocial video games on prosocial behaviors: International evidence from correlational, longitudinal, and experimental studies [49] (comm.

Total variance explained			
Component	Total	% Of variance	% Cumulative
1	4.121	58.877	58.877
2	1.114	15.914	74.791
3	.804	11.483	86.275
4	.690	9.857	96.132
5	.150	2.139	98.271
6	.090	1.281	99.552
7	.031	.448	100.000

Table 5: PCA variance explained

"media & video games")
5) The effects of violent video game habits on adolescent hostility, aggressive behaviors, and school performance [48] (comm. "media & video games")
6) The effect of video game violence on physiological desensitization to real-life violence. Journal of experimental social psychology [26] (comm. "media & video games")
7) Short-term and long-term effects of violent media on aggression in children and adults. Archives of pediatrics & adolescent medicine [21] (comm. "alcohol and aggression". 5th smallest community)
8) Testosterone responses to competition predict future aggressive behaviour at a cost to reward in men [27] (comm. "testosterone and aggression")
9) Chewing on it can chew you up: effects of rumination on triggered displaced aggression [20] (comm. "displaced aggression")
10) Chronic violent video game exposure and desensitization to violence: Behavioral and event-related brain potential data [8] (comm."media & video games")

7.4 Section D: Additional Analysis

The metric Density MNC (DMNC) was also calculated. However, after performing a Principal Component Analysis (PCA) we excluded this metric from our composite score. The inputs of the PCA were all the metrics used to calculate the composite score, with the addition of the DMNC.

Performing a PCA (Fig. 6) revealed two principal components where the first component included all the metrics taken into account, except for DMNC, whereas the second was composed by DMNC alone. Hence, it emerged that DMNC does not correlate with the other metrics and it creates a separated component. Table 5 reports the total variance explained with the extraction method of PCA.

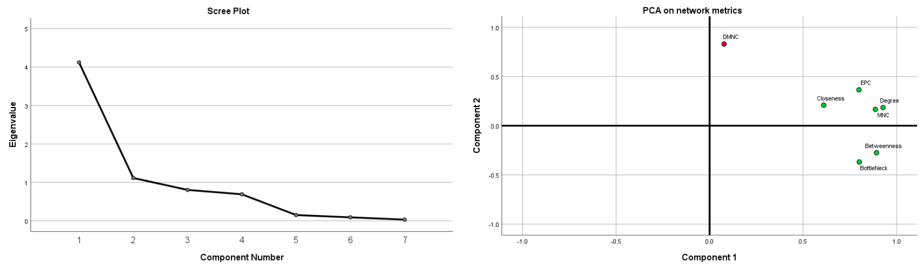

(a) Scree plot

(b) PCA analysis

Figure 6: Components. a) shows an elbow which indicates the presence of two factors. b) shows the two factors (in red and green). DMNC is the only element of the second factor

7.5 Section E: Metrics

In order to detect the most relevant papers among each community, we calculated a series of network metrics that were detailed in Table1. Here, we detail the results obtained for each metric further. The metric Betweenness centrality allows us to count the number of times a node lies on the shortest path between other nodes, and it shows which nodes (in our case papers) are 'bridges' between nodes in the network. For instance, the paper with highest Betweenness (excluding the source paper), is the paper "Atypical empathic responses in adolescents with aggressive conduct disorder: A functional MRI investigation" [34]. This paper, since it has the highest Betweenness score, controls most of the information flow in the aggression network, and it is therefore a critical point of the network. The most relevant nodes, according to the metric Betweenness, are represented in Fig. 7a (colored in orange).

We also calculated the measure of Degree centrality. Degree centrality assigns a score based solely on the number of links held by each node. This metric, in our specific case, tell us the total number of citations a paper holds. In other words, this metric shows how many times a paper has been cited by and cites other papers. For this metric, the most relevant paper is "Violent video game effects on aggression, empathy, and prosocial behavior in eastern and western countries: a meta-analytic review" [6] (excluding the seed), which means that this paper has the highest number of connections in the network with other papers. The most important nodes, according to the metric Degree, are shown in Fig. 7b (colored in sky-blue). Another measure of centrality that we took into consideration is Closeness. This measure assigns each node a score based on its sum of shortest paths. Basically it tells us how close is a given node to all other nodes in the network. For instance, papers that directly cite the seed paper, are more central than papers that cite indirectly the seed paper (second generation papers) in our human aggression network. The paper highest in closeness is "The influence of media violence on youth" [4]. This means that this paper is

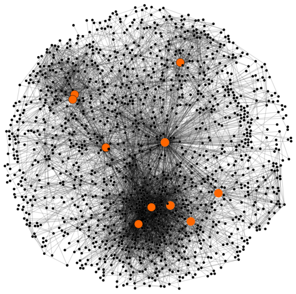

(a) Betweenness network.

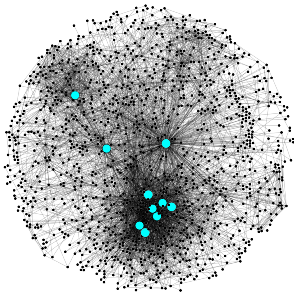

(b) Degree network.

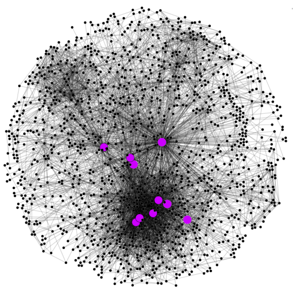

(c) Closeness network.

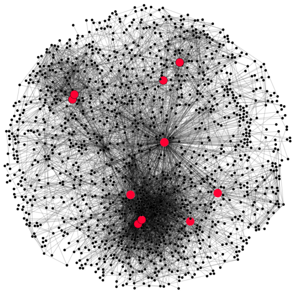

(d) bottle neck network.

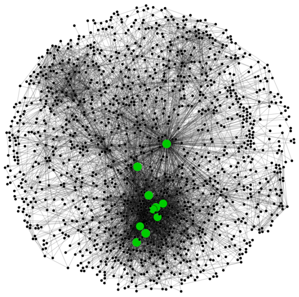

(e) EPC network.

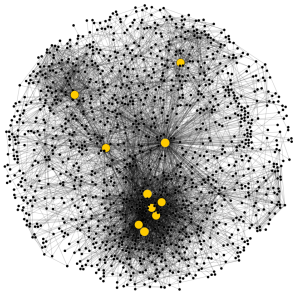

(f) MNC network.

Figure 7: Network metrics. Each figure shows the top 10 papers of a specific metric.

best placed to influence the entire network most quickly, and therefore, in our case, closeness estimates how fast the flow of information would be through a given paper to other papers. The top 10 nodes for closeness are represented in Fig. 7c (in purple).

In addition, we took into consideration the Bottle Neck metric. The red nodes in Fig. 7d are the most relevant papers according to the Bottle Neck metric. The most relevant papers for the Bottle Neck metric is again "The influence of media violence on youth" [4] (first ranked for closeness as well). As we saw in the method section, Bottleneck nodes are like choke-points in the network and have been described as being analogous to major bridges and tunnels on a highway map [112]. In our case, nodes with high bottleneck coefficient indicate papers that are essential in connecting different modules in the network, by facilitating the information flow. Furthermore, we calculated the Edges Percolated Component (EPC). The top one paper (excluding the source) for EPC is "Violent video game effects on aggression, empathy, and prosocial behavior in eastern and western countries: a meta-analytic review"[6], which is also the paper with highest degree centrality. Other papers with high EPC are "Correlates and consequences of exposure to video game violence: hostile personality, empathy, and aggressive behavior" [7] and "Prosocial Behaviors: International Evidence From Correlational, Longitudinal, and Experimental Studies" [49]. These papers are essential for keeping the structure of the graph in place. If we remove these papers, a great amount of nodes will become isolated (without any connections with other papers) and the information flow would be interrupted (Fig. 7e, relevant nodes in green).

Moreover, the maximum neighborhood component (MNC) was calculated (Fig. 7f, nodes in yellow). The paper with highest MNC is, once again Anderson (2010) [6]. This metric allows us to understand which papers have a highly connected paper within their neighborhood. Put in a simple way, if this was a Facebook network and our account was a node in the network, this metric would have told us who among our friends had the highest number of Facebook contacts.
