# Supplementary material for: Using citation network analysis to enhance scholarship in psychological science: A case study of the human aggression literature
Source: PLoS One. 2022 Apr 21;17(4):e0266513. doi: 10.1371/journal.pone.0266513 (PMC9022888; doi:10.1371/journal.pone.0266513)
Supplement: Attachment — Submitted filename: PONE-D-21-20644_comments.pdf [file pone.0266513.s002.pdf]

PONE-D-21-20644_reviewer

Title: A Network Approach to the Human Aggression Literature

General comments

This manuscript applies a community detection algorithm to analyze and identify influential papers per community of a citation network in the human aggression literature. It also analyzes possible gender biases of influential papers if communities of subfields are not considered.

Overall, I found the paper sound and original in the sense of applying well-established methods of network analysis to a particular citation network. However, before recommending for publication, I would like the authors to do a minor revision following the comments below.

Detailed comments

Introduction

- The narrative around the motivation for this study sometimes sounds like the method developed in the paper (and the problem of increasing publication volume) is specific to the aggression literature. It would sound better to pose the question in these lines: "How are scientists able to develop their expertise in a particular field, given the volume of research being published? Here, we propose a strategy – applied to the human aggression literature – to help researchers to acquire a breadth of understanding of the field of interest."
- Scientometrics (and citation networks in science) is yet another field that has increasing publication volume. The paper lacks engagement with the literature. How does your methodology fit in the literature of citation networks? Are you applying methods seen elsewhere? Are you adapting the methodology? How does it differ from others in the literature? I would like to see engagement with the literature other than "previous works have used citation network analysis to explore diverse scientific fields..."
- Paragraph 4. What biases and advantages? One example is the "positive bias for contemporary studies". However, I think a deeper reflection on possible biases and advantages might be helpful to other researchers that might want to use this method in their particular fields.
- Paragraph 4. How was the seed paper selected? Is it a subject choice based on one's knowledge of the field? The goal of the strategy proposed here is to help new researchers in the field navigating the literature. How can this new researcher choose the seed paper? How the results would change if another influential paper were selected. It would be good if the authors could test the robustness of the method subject to the choice of the seed paper.

Methodology

Section 3.1

- This is more of a reflection: Why just the first and second generation papers in the network? Couldn't it be the case that one of the poorly cited papers that were filtered out was just not cited by these two generations? That is, couldn't such a paper be opening an entirely new avenue, in a way that papers citing it do not cite the others in these two generations neither the source?

Section 3.2

- The third and fourth paragraphs of section 3.2 are confusing. Please standardize the notation and acronyms, and change the text to improve clarity.

Results

Section 4.1

- Have you tried other community detection methods? It would be nice to test how robust the results are subject to that as well.
- Figure 2. Would you please inform which algorithm was used for the layout of the network (force-directed?)? Please clarify if the size of the nodes is only to highlight the most influential ones. That is, are all other nodes of the same size?
- The first paragraph of page 9 is confusing and has repeated sentences. Please edit and clarify.

Section 4.2

- Table 3 seems to have no reference in the text where it appears. You only talk about this table in Section 5.

Section 4.3

- Fig 3 seems unnecessary, thanks to the total overlap between the bridging papers and the most influential by community, such that we can't extract any meaningful information from it. The paper doesn't explore this figure either, and Fig 2 already does the job.
- Do the results around more connected/isolated communities tell more about the field in general? I might have missed it, but I think there is no discussion about this even later on in Section 5.

Section 4.3.1

- I am a little skeptical with Figure 4. Please provide confidence interval and p-value.
- This entire section is somewhat confusing. Please rewrite to make your point (at the very end of the section) clearer.
