# Supplementary material for: Using citation network analysis to enhance scholarship in psychological science: A case study of the human aggression literature
Source: PLoS One. 2022 Apr 21;17(4):e0266513. doi: 10.1371/journal.pone.0266513 (PMC9022888; doi:10.1371/journal.pone.0266513)
Supplement: Attachment — Submitted filename: Response_to_Reviewers.pdf [file pone.0266513.s003.pdf]

Reviewer #1:

1) The narrative around the motivation for this study sometimes sounds like the method developed in the paper (and the problem of increasing publication volume) is specific to the aggression literature. It would sound better to pose the question in these lines: "How are scientists able to develop their expertise in a particular field, given the volume of research being published? Here, we propose a strategy – applied to the human aggression literature – to help researchers to acquire a breadth of understanding of the field of interest."

We agree with the reviewer on this point and we have made many changes to address this suggestion. We have revised the title to: “Using Citation Network Analysis to Enhance Scholarship in Psychological Science: A Case Study of the Human Aggression Literature” which we think does a better job of making it clear that the aggression literature is just one case study of our more general citation network analysis approach. We have also made numerous changes throughout the manuscript to follow suite including in the abstract, introduction, and discussion sections. For instance, we now include separate subheadings in the introduction and discussion to discuss citation network analysis separately from its application to the aggression literature. We also add several additional discussion points about citation network analysis specifically - in part to address many of the comments below. We appreciate the feedback from the reviewer here and believe that the resulting manuscript may be more impactful for a wider range of communities.

2) Scientometrics (and citation networks in science) is yet another field that has increasing publication volume. The paper lacks engagement with the literature. How does your methodology fit in the literature of citation networks? Are you applying methods seen elsewhere? Are you adapting the methodology? How does it differ from others in the literature? I would like to see engagement with the literature other than "previous works have used citation network analysis to explore diverse scientific fields..."

We agree with the reviewer’s comment. In the revised manuscript, we more thoroughly discuss how our study relates with other work on citation network analysis. First, for scholarship, we have now included citations to early work on citation network analysis since the 1960s (e.g. Price De Solla, 1965; McGervey 1974; Wade, 1975; Narin, 1976; White, 1977; Baker 1990; Garfield, 1964). Second, we included a new subsection in the introduction entitled “Citation Network Analysis“ which compares and contrasts our approach with prior approaches. Third, we included a new subsection in the discussion entitled “Guidelines for Future Usage” which compares our approach with other approaches for developing a citation matrix.

3) Paragraph 4. What biases and advantages? One example is the "positive bias for contemporary studies". However, I think a deeper reflection on possible biases and advantages might be helpful to other researchers that might want to use this method in their particular fields. How was the seed paper selected? Is it a subject choice based on one's knowledge of the field? The goal of the strategy proposed here is to help new researchers in the field navigating the literature. How can this new researcher choose the seed paper? How the results would change if another influential paper were selected. It would be good if the authors could test the robustness of the method subject to the choice of the seed paper.

In the revised manuscript, we now make this point more explicit and discuss the issue raised by the reviewer in more detail. In the introduction subsection entitled, “Application to Research on Human Aggression” we now provide more thorough justification for our seed-based approach in paragraphs 2 and 3 of that subsection, which is oriented toward our goals of unpacking the aggression literature. We highlight how several criteria are informative for our seed selection: journal (Annual Review of Psychology), publication date, citations, etc.

In the revised discussion section, we reflect on taking a seed-based approach more generally (i.e. not in particular for research on human aggression) for researchers that might want to use this method for their own disciplines. These changes are described in the newly added subsection entitled “Guidelines for Future Usage”. We further discuss in that subsection how seed selections can rely on qualitative and quantitative criteria, and also the strengths and weaknesses of using a seed based approach for developing a citation matrix relative to other approaches (e.g. topic or keyword searches).

When taking a seed-based approach, seed selection will likely have a tremendous impact on the outcome of the results. For example, a much more niche article (by definition) will only be cited by articles from a single community, leading to a different and much more limited solution. Seed selection is, therefore, critical. Running another seed might show overlapping but also unique communities. It stands to reason that researchers could, if they’d like, run the analysis using multiple seeds and identify multiple network structures. Doing so might help identify both shared communities and also unique communities from each seed. Inferentially speaking, if communities are observed from one seed that aren’t observed from another seed, the lack of robustness in that case does not necessarily mean that those communities are not of relevance or importance. In that sense, comparisons of seeds is not necessarily a window into robustness - each seed will likely provide different topological information about the literature at large.

We note that at least for the literature on human aggression, the article we selected truly stands out - there may not be a clear alternative choice for selecting a seed. We aimed to

select a seed that provided a major comprehensive review of the field (as from the Annual Review series) which will thus capture at least many relevant topics in the field, was about two decades old (which will thus contribute to a citation matrix of papers that cited the seed that is more contemporary), was highly cited (and thus will help develop a large citation matrix on the field), and peer-reviewed. Other potential contenders included books that, while being highly cited, were not peer-reviewed, focussed on specific perspectives, and were published long ago. In the human aggression literature, there were no other articles of the same class.

That being said, not every literature will have these same qualities. In future studies, it would be interesting to compare the solutions of distinct solutions networks generated from diverse seeds. To be sure, this would require developing formal computational methods for comparing heterogeneous networks - which currently falls outside the scope of our work (and poses many unique challenges given the temporal dynamics of publications). In the revised manuscript, we now describe these issues in more detail in the subsection "Guidelines for Future Usage", and we offer the suggestion to users to select multiple seeds papers, given their goals.

4) Section 3.1 This is more of a reflection: Why just the first and second generation papers in the network? Couldn't it be the case that one of the poorly cited papers that were filtered out was just not cited by these two generations? That is, couldn't such a paper be opening an entirely new avenue, in a way that papers citing it do not cite the others in these two generations neither the source? *"Couldn't it be the case that one of the poorly cited papers that were filtered out was just not cited by these two generations?"*

We appreciate these reflective questions!

First, we note for the papers that were filtered out, these articles were filtered out based on total citations throughout the entire literature (not just the number of citations within the 1st and 2nd generation articles of the aggression matrix). The vast majority of the papers filtered out had zero, total citations. These articles are essentially leafs or isolated nodes in a network and are often dropped from citation network analysis. We clarified this point in the revised manuscript (in the revised Methods section) where we now indicate that we excluded articles with 2 or fewer total citations.

Second, it could be the case that some of those papers that were filtered out were too young to be cited. It would be interesting to re-run the analysis in 5 years to see the evolution of the network and the emergence of new communities. We have added this point as a limitation in the discussion section, where we state: "Finally, our matrix included only 2

generations, and so the results are limited in addressing the prospective contributions of younger articles. It would be interesting to re-run the analysis in the future to see the evolution of the network and the emergence of new communities.”

Third, it is possible that there are papers that were included in the citation network analysis, but their contributions were overlooked since we stopped our matrix at 2 generations. In other words, there might be papers that were cited many times in the wider literature, but were cited only a few times in the network. Speculatively, these papers could be bridges to new communities that were not included in our particular citation network analysis because they (collectively) rarely cited the seed article or 1st generation articles that were in our matrix. This might be possible, albeit we think it is unlikely that those communities would be clearly linked to the human aggression literature - or if they are, they are linked in a way that appears to have disconnected itself with a rich history of scholarship in the field. If we expanded to a third or fourth generation, we may also start pushing the boundaries to literature that eventually has little to do with human aggression.

Section 3.2

5) The third and fourth paragraphs of section 3.2 are confusing. Please standardize the notation and acronyms, and change the text to improve clarity.

The old section 3.2 has been rewritten to improve clarity and it is now included in the new subsection “Citation Network analysis” in the methodology section. We standardized the notation and acronyms as well. In addition, we included the rationale for the algorithm that we selected for our community detection analysis, and we also included a new analysis on community interconnectedness.

Results

Section 4.1

6) Have you tried other community detection methods? It would be nice to test how robust the results are subject to that as well.

We can see how our justification for using the community detection approach was insufficient in our prior manuscript. While there are many different community detection algorithms, we opted to use the Louvain algorithm for community detection. Other community detection algorithms (e.g. those that were based on link centrality) have a high computational cost, which would have been problematic especially for the network validation method we used (i.e. NMI with subsampling). A more suitable family of algorithms for our goal is the modularity optimization one. There were two candidates from this family

including the “Fast Greedy” algorithm and Louvain. The latter was preferable because it includes a community aggregation step to improve processing on large networks (Blondel, 2008). Our community detection method is, thus, particularly well suited for our goal of providing readers with an easy tool to use to characterize a literature. We now provide further justification for our methods in the revised manuscript (Subsection “citation network analysis” of the methodology section):

“There exists a considerable amount of community detection algorithms, yet, the Louvain algorithm was preferable in our case because it includes a community aggregation step to improve processing on large networks (Blondel, 2008). Conceptually, ``communities” are formed from groups of papers that tend to be cited by the same papers. This algorithm maximizes a modularity score for each community; specifically, it compares how much more densely connected the nodes within a community are with how connected the nodes would be in a random network [12].”

While our aim was not to conduct a comparison of different algorithms per se (albeit we can do so if the reviewer requests additional justification than provided above), robustness and performance can be obtained by estimating the community structure of reference with techniques such as Normalized Mutual Information (the one that we used). In our work, we did indeed validate our network architecture over 2000 times by applying the community Louvain algorithm to a subset of 90% of randomly selected nodes. Then we used Normalized Mutual Information (NMI) to evaluate the consistency of solutions, which was 0.64 (1 = perfect consistency).

7) Figure 2. Would you please inform which algorithm was used for the layout of the network (force-directed?)? Please clarify if the size of the nodes is only to highlight the most influential ones. That is, are all other nodes of the same size?

We edited Figure 2 to clarify these issues. We now note that the size of the nodes highlights only the most influential papers for visualization purposes, whereas the rest of the nodes are all represented with the same size. The layout used to produce this visualization is “Perfuse Force Directed Layout”.

8) The first paragraph of page 9 is confusing and has repeated sentences. Please edit and clarify.

We removed the repeated sentence in the paragraph. In addition, we rewrote the “community detection” subsection in the result section to improve clarity. To ease the burden of the review process, we paste below the relevant text from the edited section:

> “It is useful to compare our findings from the community detection analysis (which focuses on clusters of papers) with citation ranking and graph theoretic measures (which focus on

individual papers) irrespective of community. The top 10 papers based on citation ranking and based on our composite measure of influence are presented in the supplementary material (section C) and Table 4 (also see supplementary material, section A), respectively. Strikingly, with the exclusion of the seed paper, no paper from the second biggest community appears among the top 10 most cited articles nor in the top 10 most influential papers from our composite ranking. Indeed, 7 of the top 10 papers based on citation ranking were all from a single community. Altogether, these findings highlight the usefulness of the clustering approach in uncovering the community-based topology of a research field (Table 3), and our composite ranking score in identifying key articles for each community (Table 2)."

Section 4.2

9) Table 3 seems to have no reference in the text where it appears. You only talk about this table in Section 5.

Table 3 has now two references in the text in the result section (subsection "Community Detection"), and one in the discussion section.

Section 4.3

10) Fig 3 seems unnecessary, thanks to the total overlap between the bridging papers and the most influential by community, such that we can't extract any meaningful information from it. The paper doesn't explore this figure either, and Fig 2 already does the job.

Figure 3 from the original manuscript has been removed.

11) Do the results around more connected/isolated communities tell more about the field in general? I might have missed it, but I think there is no discussion about this even later on in Section 5.

We can see how the original interconnectedness analyses were not quite informative about the literature or for our goals. Upon reflection on this point, we have performed a new interconnectedness analysis that estimates how close two communities are to one another based on the average shortest paths of nodes between communities. We have revised the methods and results sections to describe this new analysis. We also outline the key findings in the discussion section, and we introduce a new Figure 3 to illustrate the interconnectedness of communities. We believe this provides unique insights on the topology of the aggression literature by neatly illustrating how close or far apart different communities are to one another.

To ease the burden of review, we include the new relevant text here:

Methods:

> "Fourth, we examined the extent to which communities are interconnected. To do that, we calculated the average shortness distance between each community pair. Subsequently, we inputted the distance matrix, containing the pairwise distances between communities, to Multidimensional Scaling (MDS) in order to visualize the level of similarity between communities."

Results and Discussion:

> "We used a measure of interconnectedness to determine which communities were closer and further from each other. Pairwise interconnectedness scores by communities were submitted to Multi Dimension Scaling for visualization. As shown in Figure 3, media and videogames, rumination and displaced aggression, and stress traits and aggression appear to be more interconnected with one another, suggesting that scholars are to some extent aware of the work done across these communities. Conversely, social pain and exclusion, testosterone, PTSD as well as oxytocin are relatively more isolated communities. Aggression in horses was the most isolated community."

> "We produced the first citation-based literature map of research on human aggression, our topic of interest. Our analysis identified 15 research communities on aggression, how interconnected these communities are with one another, the top five influential papers within each community using a composite of graph-theoretic measures, and papers that bridge communities - which happened to be the same as the most influential papers for each community."

12) I am a little skeptical with Figure 4. Please provide confidence interval and p-value.

Given several of the points made by the reviewers, we have made several revisions to the manuscript to deliver a more coherent manuscript. In doing so, we found that Figure 4 was actually unnecessary and distracting for the main points of the paper. In any case, for transparency with the reviewer on this point, the stats regarding the old figure 4 were the following: $R = 0.6330$; $P < 0.0001$; 95% CI: RLower = 0.5998, RUpper = 0.6640

13) This entire section is somewhat confusing. Please rewrite to make your point (at the very end of the section) clearer.

To improve clarity, we divided the old paragraph 4.3 ("Bridging papers and community inter-connection") into two subsections ("Bridging Papers" and "Community Interconnectedness" respectively). We also performed different Interconnectedness analysis based on the average

shortest path among communities. Two easy the burden of the review process, we paste the text from these new two subsections below:

"Bridging Papers" :

"So far, our analysis identifies certain research communities and the most influential papers for each community. It may also be useful to know which papers in a given community serve as bridges to other communities. Bridge nodes may be important for information exchange across communities throughout a network (Liu, 2019). Conceptually, bridge nodes are papers that are often on the shortest path between papers (i.e. have high betweenness centrality) and that are cited by other papers from different groups (i.e., communities). Importantly, since our citation network is directed, a bridge node is one that is connected through unidirectional links (i.e., "cited by" relationship). In our directed graph, we found that the top five bridging nodes for each community ended up being the same as the five most influential papers of that community (Table 3). To be sure, this overlap does not necessarily reflect an inherent relationship between the influence metric and the bridging metric - it is likely that for other citation network analyses, bridging papers may not overlap with influential papers within a community. However, for newcomers to the human aggression literature, this overlap can be viewed as helpful insofar as it helps constrain the subset of papers that one might select for developing scholarship. "

"Community Interconnectedness"

We used a measure of interconnectedness to determine which communities were closer and further from each other. Pairwise interconnectedness scores by communities were submitted to MDS for visualization. As shown in Figure 3, media and videogames, rumination and displaced aggression, and stress traits and aggression appear to be more interconnected with one another, suggesting that scholars are to some extent aware of the work done across these communities. Conversely, social pain and exclusion, testosterone, PTSD as well as oxytocin are relatively more isolated communities. Aggression in horses was the most isolated community.

Reviewer #2:

1) In the chosen network analysis metrics there was little consideration to the directed nature of the networks. This is especially important in the case of the bridging nodes. The authors use an algorithm for finding bridging nodes that

consider an undirected network. Following this disregard for the directed nature of the network, the conclusions from the results assume a bi-directional network. The authors say that "the top five papers in the top five communities are not only the most influential within their communities but they are also connecting papers between communities in the human aggression network". This conclusion is valid only for an undirected network. For a directed network, I would imagine that these papers, building on "older" communities, maybe found a new angle that became the basis for the newer community. Please see to discuss the effect of the network being directed on the bridging algorithm and reconsider your conclusions in this regard.

The reviewer suggests that we overlooked the directed nature of the network, and, as a consequence, that the conclusions we provide are valid only for an undirected network. Upon further reflection, we understand that in the original version of the manuscript we over-interpreted the results of the bridging nodes. In the edited version of the manuscript, we addressed the reviewer concerns by revising the entire subsection dedicated to the bridging nodes. We mitigated some of our conclusions that assume a bi-directional network. At the same time, prior work in network analysis has used certain graph theoretic measures designed for undirected graphs but on directed graphs. In such cases, it is important to be clearer about the meaning of these analyses. Thus, we have revised the section on bridging nodes to reflect this concern. The revised manuscript now states:

> “So far, our analysis identifies certain research communities and the most influential papers for each community. It may also be useful to know which papers in a given community serve as bridges to other communities. Bridge nodes may be important for information exchange across communities throughout a network (Liu, 2019). Conceptually, bridge nodes are papers that are often on the shortest path between papers (i.e. have high betweenness centrality) and that are cited by other papers from different groups (i.e., communities). Importantly, since our citation network is directed, a bridge node is one that is connected through unidirectional links (i.e., “cited by” relationship). In our directed graph, we found that the top five bridging nodes for each community ended up being the same as the five most influential papers of that community (Table 3). To be sure, this overlap does not necessarily reflect an inherent relationship between the influence metric and the bridging metric - it is likely that for other citation network analyses, bridging papers may not overlap with influential papers within a community. However, for newcomers to the human aggression literature, this overlap can be viewed as helpful insofar as it helps constrain the subset of papers that one might select for developing scholarship. ”

2) The directed nature of the network implies also temporal ordering. This means that younger papers have less chance of being cited than older ones. Is it possible to control for this in some way? I am not from the Regression world but would it be safe to say that the size of some of the communities correlated with their age? If so, an interesting thing to find is "growing communities" vs. "stable communities". One might think of it maybe as "trendy communities" = find for each community the fraction of the number of new citations to their size, and see which is the fastest growing and whether there are communities that are starting to become less appealing?

To address the reviewer question concerning a possible correlation between communities' size and age, we performed a correlation analysis between the communities' size and their date of origin in the network. Surprisingly, this correlation ($r = - .048$), even though it was moderate and negative (the smaller the community, the younger -higher year-) it did not reach significance ($p = 0.1$).

Furthermore, the reviewer also expresses interest in the temporal growth of the network. We were very excited to follow this suggestion and we thank the reviewer for this excellent remark! Hence, we provided an initial examination of the growth tendencies of the main communities in the human aggression network. The results are illustrated in a new subsection "Community Growth", in the results section. Here, we were able to identify the fast-growing communities by size (number of publications) and by influence (number of citations).

While it is beyond the scope of this work, it would be very interesting in future studies to follow our network in a longitudinal way to better understand the evolution of the human aggression field. Will a particular community emerge in 5 or so years? Will new papers serve as bridges for communities that were not communicating before? Unfortunately, we cannot answer these questions in the present manuscript, and we acknowledge the limitation this places on younger papers. To ease the burden of the review process, we pasted below the relevant text from the new subsection "Community Growth".

(Community Growth -in the results section):
"Next, we investigated how communities grew in size and influence over time. That is, we plotted the quantity of publications and total number of citations across years per community. The boundary of our time window, given our citation matrix, is from 2002-2019 and so interpretation of findings should be understood as contingent on this temporal boundary. Further, interpretation should also be contingent on our seed-based approach of developing the citation matrix. We focus our description primarily on the major trends that are observable from the results. Figure 4 displays the findings. Naturally, the number of citations trailed the number of publications for each community. The three largest communities - ``media and video games", ``stress traits and aggression" and ``rumination and displaced aggression" - have steadily grown over time with peaks around 2015-2016 in volume and around 2017 in total citations. The two smaller of the top five communities - ``testosterone" and ``social aggression" - appeared to start their growth later in time but peaked at around the same time."

2) Another thing that I have noticed is that about third of the prominent papers depicted in Figure 7 in the supporting information came from the largest community (apart from the case of figure 7d, the bottleneck papers). Is this community the "Media and Video games"? It is the largest in the network, and the denser. It is easy to conclude that high degree nodes are then highly cited within the community and also from other communities, hence have high degree and also high closeness (and of course high EPC and high MNC).

It should then be noted that the choice of having only two levels of distance from the seed node caused an overlap in the majority of the chosen metrics.

- Please refer in your text to the points of overlap in the metrics due to the structure of the network and the resulted overlap in some of the results, especially for prominent papers.

The reviewers expressed concern on a possible overlap on the majority of the metrics that we selected to calculate our composite score. We agree with the reviewer's points and have revised the manuscript accordingly. In the last section of the discussion "Conclusion and Future Directions", we now state:

"A possible limitation of this analysis could be that we only took into consideration the papers that cite the seed articles (first generation) and the papers that cite the first generation articles. Therefore, our network is composed of two levels of distance from the seed node. This structure of the network is likely to have produced an overlap in several metrics that we have chosen to calculate our composite scores and thus the communities. For instance, for several nodes (especially for the nodes with a larger number of edges) the value of betweenness centrality was very similar to the value of closeness. Our constraints in generating the citation matrix could have made it difficult to distinguish communities, too. Nevertheless, the analysis identified several distinct communities, suggesting that these constraints may not be overly restrictive in uncovering the community structure of a field."

3) Lastly, the entire methodology relies on your understanding of the research area, by choosing a seed paper. This could be the reason to some of the limitations you discuss. I suggest to discuss alternatives to the seed approach in the Methodology Section and their implications.

We now discuss alternative methods to seed papers (e.g., keyword network analysis) and their implications in the discussion section (new subsection "Guidelines for Future Use"). To ease the burden of the review process, we paste the relevant text below

"A citation matrix can also be developed using keywords to identify articles and using citations to link the articles. A citation matrix defined from keywords may reveal communities

that actually have no link to one another at all (whereas in seed-based approach every article is eventually linked to every other article in the network at least through the seed). Going one step further, keyword similarities can also be used as edges to link articles to one another instead of using citations (Ding, 2001; Kim, 2021; Choi and Hwang, 2014) or even using the full-text to identify topics and communities (e.g., Liu et al., 2014). However, analysis using keywords or particular terms introduces its own set of challenges. For example, keywords can be expressed in different ways by the authors (e.g. synonyms), and so identifying relevant structures amongst keywords may require field expertise.

Overall, we view these methods as complementary approaches in the broader family of literature network analysis. It may be of interest to future work to utilize a variety of different approaches to more formally compare and contrast them against each other and see how it changes the network model. Ultimately, however, the method one chooses depends on the goals of the researcher. Technically, our graph theoretic analysis can be flexibly applied to literature matrices that use a seed-based, keyword, or full-text approach. And our seed-based approach is particularly useful when expertise is minimal, so long as a seed can be identified perhaps by using the aforementioned strategies. Of note, it is also useful from a historical perspective for tracing the contributions of a particular line of work or author."

.

In addition, we integrated our introduction with the advantages and disadvantages of choosing a seed paper, along with potential biases. That is, in the introduction subsection entitled, "Application to Research on Human Aggression" we now provide more thorough justification for our seed-based approach in paragraphs 2 and 3 of that subsection, and we highlight how several criteria are informative for our seed selection: journal (Annual Review of Psychology), publication date, citations, etc.

In the revised discussion section, we also reflect on taking a seed-based approach more generally (i.e. not in particular for research on human aggression) for researchers that might want to use this method for their own disciplines. These changes are described in the newly added subsection entitled "Guidelines for Future Usage". We further discuss in that subsection how seed selections can rely on qualitative and quantitative criteria as well.

The relevant text is pasted below.

"More generally, seed papers can be selected via quantitative criteria such as centrality, citations, journal impact factor, or by using qualitative criteria (e.g., expertise of the research team, perceived journal quality, reputation of a lead author) or a combination of both criteria (Wang, 2021). Each approach has strengths and limitations that will ultimately impact the citation matrix (Donner, 2018; Onodera, 2015; Xie, 2019; Haslam, 2010; Hegarty, 2012; Nickerson, 1998).

> [...] Specifically, users may consider selecting a seed article using the same rationale that we outlined above: a highly-cited, peer-reviewed, comprehensive review article as are commonly published in certain outlets (e.g. the Annual Review series). To be sure, exactly which features matter will depend on the goals of the researcher in conducting their literature review."

All the remaining minor remarks from the previous review have also been addressed in the revised manuscript.

We included a step-by-step commented code (starting from data gathering up to the visualization of the network) as well.

Link: [GitHub - ABS-Lab/A-Network-Approach-to-the-Human-Aggression-Literature: Accesss to the data used in the paper"A Network Approach to the Human Aggression Literature"]
